# Bio-syncretic robots composed of biological and electromechanical systems

**DOI:** 10.1093/nsr/nwac274

**Published:** 2022-12-02

**Authors:** Chuang Zhang, Jia Yang, Wenxue Wang, Lianqing Liu

**Affiliations:** State Key Laboratory of Robotics, Shenyang Institute of Automation, Chinese Academy of Sciences, China; Institute for Robotics and Intelligent Manufacturing, Chinese Academy of Sciences, China; State Key Laboratory of Robotics, Shenyang Institute of Automation, Chinese Academy of Sciences, China; Institute for Robotics and Intelligent Manufacturing, Chinese Academy of Sciences, China; University of Chinese Academy of Sciences, China; State Key Laboratory of Robotics, Shenyang Institute of Automation, Chinese Academy of Sciences, China; Institute for Robotics and Intelligent Manufacturing, Chinese Academy of Sciences, China; State Key Laboratory of Robotics, Shenyang Institute of Automation, Chinese Academy of Sciences, China; Institute for Robotics and Intelligent Manufacturing, Chinese Academy of Sciences, China

## Abstract

Potential approaches to developing bio-syncretic robots are proposed in different dimensions, including monomial functions and closed loop systems of living cell-based sensing, intelligence, and actuation.

After eons of evolution, biological systems have developed excellent performance in terms of sensing, intelligence, and actuation—the three primary robotics elements that are closely related to the overall performance of robot systems. For example, the buccal pits of some snakes, as the necessary sensors for accurate predation, have a high-temperature sensitivity of 0.003 K [1]; the biological brain can exhibit an excellent capacity for efficient learning and multitasking operations [2]; and froghopper insects can leap to a height of more than 100 times their body length [3]. Most of these biological system performances are difficult to realize with traditional electromechanical systems. Electromechanical systems also have some advantages, such as high accuracy, high strength, high power, and favorable repeatability, which may be scarce for biological systems. Therefore, to effectively improve robot performance, bio-syncretic robots comprising biological and electromechanical systems at the molecular, cellular, and tissue levels have been proposed in recent years. Such robots directly utilize biological materials as their functional cores and may realize the complementary advantages of biological and electromechanical systems [4].

Due to their considerable advantages, bio-syncretic robots have drawn much attention, and the field has become a research hotspot. In terms of sensing, among the biological sensing functions, visual sensing may occupy up to 80% of the external information received by humans; therefore, bio-syncretic visual sensing has been widely researched. Some photosensitive biological materials, such as bacteria and cells, have been used to realize the effective transformation of environmental information into electrical signals based on the electrophysiological response under external stimulation. Moreover, by integrating biological materials with artificial electronic systems, various bio-syncretic sensing systems have demonstrated effective environment sensing [5,6]. Regarding intelligence, distinct neural networks derived from human pluripotent stem cells or isolated from the brain tissue of rats have been cultured *in vitro* and were combined with silicon computing systems with microelectrode array (MEA) chips to form a bio-syncretic intelligence controller. Living neural networks can be dynamically trained in control processes to realize effective learning and control of simulated and physical robots [7,8]. For actuation, various contractile living materials, such as cardiomyocytes, skeleton muscle cells, and dorsal vascular tissues of insects, have been used as actuators and subsequently integrated with artificial structures to construct bio-syncretic robots with different locomotion modalities, including swimming, walking, and manipulation, etc. In addition, robot control methods based on different mechanisms, such as electrical or optical stimulation, have been explored to realize the controllability of bio-syncretic robots [9,10].

Existing research on bio-syncretic robots has preliminarily verified that it is feasible to use biological materials as robotic function cores. However, the majority have only demonstrated simple functions, meaning that there is much more work to be done in the field to give full play to the functions of biological systems. Thus, further exploration of bio-syncretic robots in terms of sensing, intelligence, and actuation is necessary for their practical application. The potential roadmap for the development of bio-syncretic robots is outlined in Fig. 1.

**Figure 1.**
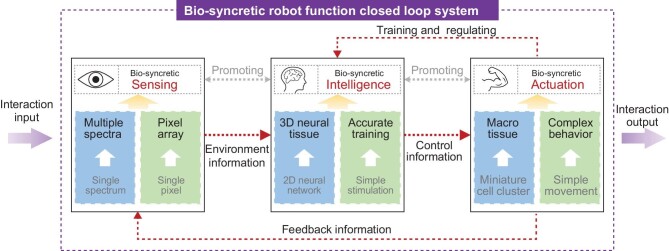
Potential roadmap for the development of bio-syncretic robots, including sensing, intelligence, and actuation functions.

## Sensing: from a single spectrum and single pixel to multiple spectra and an array of pixels

Current bio-syncretic sensors mainly focus on specific single spectrum imaging with a single photosensitive pixel, which greatly restricts sensing applications due to low sensing efficiency and sparse spectral information. To improve imaging performance, first, biological materials able to respond to multiple spectra, including visible and infrared light, should be exploited by engineering powerful variants or advancing co-transfection techniques. Second, MEA could be used to realize a sensing array that can detect the optoelectronic signals of ordered photosensitive cells cultured on it. Indeed, nanomaterials such as graphene and molybdic sulfide can be adopted to effectively enhance cellular signals. Finally, image reconstruction algorithms for multichannel cellular signals should be explored based on the imaging mechanism of humans to realize high-efficiency and multispectral imaging.

## Intelligence: from a 2D neural network and simple stimulation to 3D neural tissue and accurate training

Currently, most bio-syncretic intelligence is based on a 2D neural network, which restricts connection complexity and information processing capacity. Furthermore, the MEA used in these works only provides simple records and stimulation for the network, which may be disadvantageous for complex information transmission and accurate network training. On the one hand, increasing the intelligence level of the bio-syncretic controller may depend on an *in vitro* 3D neural network comprised of multiple layers with the capability of complex information processing. On the other hand, a matching 3D MEA should be developed to realize accurate stimulation and high-throughput information interaction with external devices. The stimulation method, comprising multiple sources, such as electrical, optical, and chemical sources, should also be researched to execute selective training of different cell types and distinct regions. It is worth noting that training with motion feedback may be a potential method to effectively generate intelligence in neural networks. The plastic connections between nerve cells will be intensively regulated by selective stimulation based on the comprehensive information of the motion targets and behaviors of the robot to realize intelligent learning and control.

## Actuation: from miniature cell clusters and simple movement to macroscopic tissue and complex behavior

Existing bio-syncretic robots mainly focus on simple motions based on *in vitro* cultured miniature cell clusters. Although the small size of the current robots is advantageous for special applications, such as medicine and detection in narrow spaces, miniature biological actuators are rarely used in macroscopic robots, thereby restricting the development of bio-syncretic robots on different scales. As such, bioprinting technology is urgently needed to fabricate macroscopic muscle actuation tissues with blood vessels to create large-sized bio-syncretic robots. Indeed, bionic integration inspired by bone and muscle tissues may be a potential approach to the effective rigid–flexible coupling of nonliving structures with the biological actuators of bio-syncretic robots. Moreover, organisms are able to perform complex and flexible behaviors depending on multiple cooperative muscle tissues. Therefore, a robot consisting of multiple biological actuators under synergetic control through artificial or biological interfaces, such as nonliving MEA, microprinted flexible electrodes, and living neuromuscular junctions, should be designed to go beyond simple movements to realize more complex behaviors.

The above in-depth study will effectively improve the performance and application of bio-syncretic function, including sensing, intelligence, and actuation. Current bio-syncretic robots with monomial functions struggle to interact with humans and the environment, while humans are able to process efficient human–human and human–environment interaction through the organic combination of sensing, intelligence, and actuation. Therefore, the closed loop of sensing–intelligence–actuation is necessary to drive the development of bio-syncretic robots from a single function to intelligent cooperative behavior (Fig. 1). In the proposed closed-loop bio-syncretic system, each function element of sensing, intelligence, and actuation can be integrated with artificial electromechanical systems by the interfaces of MEA, flexible micro wires, micro printed electrodes, optical fibers, and so on. Moreover, functional elements can also be connected by biological materials, such as nerve fibers and neuromuscular junctions. In this approach, the connection between each function module is based on biological information flow, and the nerve cells of the intelligence unit will be physiologically connected to both the sensing and actuation units. This will not only realize information transfer but also reciprocally enhance the functional performance and lifespan of the bio-syncretic units of sensing, intelligence, and actuation. Therefore, the integration of sensing, intelligence, and actuation will effectively improve the functional systematization and overall performance of bio-syncretic robots.

The proposed development of bio-syncretic robots will improve their sensing, intelligence, actuation, and interaction capabilities. Furthermore, bio-syncretic robots may be useful in other fields. For example, *in vivo* robots actuated by living cells can realize self-propelling motion with bioenergy. Moreover, bio-syncretic robots comprised of human-derived biological materials and electromechanical systems may possess preferable biocompatibility by avoiding the immune response [11]. In addition, the constructed *in vitro* bio-syncretic systems may be beneficial in better understanding living systems and in medical research and development. However, the further development of bio-syncretic robots relies on progress across multiple academic disciplines, including neuromuscular control mechanisms, robotic autonomous and wireless control methods, integrated biology–mechatronics manufacturing, and biological activity *in vitro* maintaining technologies, such as vascularized nutrition transport, microfluid chip-based medium renewal, and packaging skin-based living material protection. It is hoped that, in the future, bio-syncretic robots may realize a heightened coexisting–cooperative–cognitive working mode with humans and the environment, allowing them to serve society more safely and efficiently.
